# Prognostic impact of somatic mutations among patients with pleural and peritoneal mesothelioma

**DOI:** 10.1038/s41698-026-01495-x

**Published:** 2026-07-09

**Authors:** Justin M. Bader, Ankit Dhiman, Nicole Aguirre, Kwasi Ansere Ofori, Princy Gupta, Hannah Qin, Anup Sharma, Owen Mitchell, Melissa Y. Tjota, Aliya N. Husain, Michael Drazer, Jane Churpek, Hedy Kindler, Kiran Turaga

**Affiliations:** 1 Department of Surgery, Yale New Haven Hospital, New Haven, CT USA; 2 National Cancer Institute of the National Institutes of Health, Bethesda, MD USA; 3 Department of Surgery, Medical College of Georgia, Augusta, GA USA; 4 Yale University, New Haven, CT USA; 5 Section of Hematology/Oncology, Department of Medicine, University of Chicago, Chicago, IL USA; 6 Department of Pathology, University of Chicago, Chicago, IL USA; 7 Section of Hematology/Oncology, Department of Medicine, University of Wisconsin, Madison, WI USA; 8 Division of Surgical Oncology, Department of Surgery, Yale University, New Haven, CT USA

**Keywords:** Biomarkers, Cancer, Oncology

## Abstract

Mesothelioma is a rare cancer with poor prognosis. Somatic mutations show prognostic value in smaller studies; however, detailed survival analysis of mutations, specific variants, and co-mutations are unknown. This study gathered one of the largest mesothelioma cohorts in North America to determine prognostic impact of treatments, tumor characteristics, and somatic mutations. Among 195 patients, 70% (n = 137) had pleural and 30% (n = 58) had peritoneal mesothelioma. *NF2*, *TERT*, and *CDKN2A* had worse OS in pleural mesothelioma. *NF2* mutations with truncated NF2 protein (non-sense and frameshift with premature stop codon) had worse OS in pleural mesothelioma (log-rank-p < 0.001). Conversely, *NF2* pathogenic mutations with loss-of-function/structural variants were *not* associated with OS, revealing that *NF2* truncating mutations may drive *NF2*’s prognostic impact. This is emphasized by higher mortality at 1-year (44% vs 7.7%, p = 0.044) and 18-months (69% vs 17%, p = 0.006) compared to non-truncating *NF2* mutations. Overall, this study found that pleural and peritoneal mesothelioma have unique mutations with prognostic significance. Furthermore, as trials demonstrate varied results in *NF2*-targeted treatments, this study supports biomarker-informed strategies to guide trial enrollment and development of targeted-therapies.

## Introduction

Mesothelioma is a rare but highly aggressive cancer which most frequently originates in the pleura (~65–70%) or peritoneum (~30%)^1–3^. Despite advances in systemic chemotherapy, cytoreductive surgery (CRS), and immunotherapy, overall survival remains poor and highly variable^4–7^. Without treatment, survival is typically 6–12 months, while five-year survival remains <10% for pleural mesothelioma but can approach 20% in peritoneal mesothelioma^8–10^. For patients with peritoneal mesothelioma, this survival benefit may come from advances in treatment with select patients achieving a median overall survival of 29–98 months with CRS and hyperthermic intraperitoneal chemotherapy (HIPEC)—though outcomes are strongly influenced by histology and completeness of cytoreduction^5,11–16^. Furthermore, for patients with pleural mesothelioma, MARS2 trial emphasized the need for better patient selection for surgical cytoreduction^17^. Collectively, these observations highlight pleural and peritoneal mesothelioma as related but biologically and clinically distinct diseases.

Given the modest impact of current therapies, attention has increasingly turned toward the molecular drivers of mesothelioma. Large-scale sequencing efforts such as The Cancer Genome Atlas have identified recurrent alterations in *BAP1*, *NF2*, *CDKN2A*, and *TP53*, particularly among pleural tumors^18,19^. In peritoneal mesothelioma, homozygous *CDKN2A* deletions and *NF2* loss have been associated with worse outcomes, while *BAP1* mutations may infer a better prognosis, although these are still being investigated^20–23^. Overall, the prognostic significance of these mutations and their specific mutational variants, remains uncertain; and most molecular insights have been derived from relatively small and limited patient cohorts. Moreover, many of the major guidelines and clinical trials focus on pleural rather than peritoneal mesothelioma due to the lower incidence of the peritoneal disease site. However, this leaves peritoneal mesothelioma to be comparatively understudied despite its distinct biology, presentation, and treatment approaches^7,17^.

To date, there are very limited studies comparing pleural and peritoneal mesothelioma, which show prognostic significance for specific mutational variants and how the co-occurrence of these mutations affects survival. Furthermore, although prior studies have investigated the prognostic implication of *NF2* and *CDKN2A* mutations in mesothelioma^20,21,24^, only a few small studies in Europe have demonstrated formal survival analysis for *TERT* with none having evaluated the prognostic significance of specific mutational variants within *TERT*^25,26^.

We therefore analyzed a large cohort of patients with mesothelioma at University of Chicago to characterize the prognostic implication of somatic mutational profiles. By evaluating pleural and peritoneal mesothelioma subtypes, this study aims to clarify their distinct clinical and molecular features and to define the prognostic impact of recurrent somatic mutations. This work addresses a critical gap by linking genomic alterations with clinical outcomes in a large cohort of patients with mesothelioma.

## Results

### Patient demographics and treatment patterns

Among 199 patients diagnosed with mesothelioma between 2010 and 2022, tumor site included 69% (n = 137) pleural-only, 29% (n = 58) peritoneal-only, and 2% (n = 4) bicavitary. Most patients were female (62%, n = 123), white (97%, n = 194), and underwent non-palliative treatments (51%, n = 99). Pleural mesothelioma patients were diagnosed at an older age (68 vs 59-years-old, p < 0.001) and more likely to receive adjuvant chemotherapy (28% vs 12%, p = 0.014) and palliative-only treatment (46% vs 62%, p = 0.039) compared to peritoneal mesothelioma patients (Table 1).

**Table 1:** Demographics, treatment, pathologic findings, and survival characteristics

		Number of Patients (% of all patients)		
	All Patients	Pleural Mesothelioma	Peritoneal Mesothelioma	p-value‡
Patient Characteristics	N = 199^a^	N = 137	N = 58	
**Demographics and Medical History**				
Age at Diagnosis, years (SD)	65 (12)	68 (10)	59 (13)	**<0.001**
Sex:				0.11
Female	123 (62%)	90 (66%)	31 (53%)	
Male	76 (38%)	47 (34%)	27 (47%)	
White	194 (97%)	132 (96%)	58 (100%)	0.3
Asbestos Exposure	93 (47%)	70 (51%)	22 (38%)	0.092
History of Other Cancer	38 (19%)	24 (18%)	14 (24%)	0.3
**Treatment Received**				
Treatment Type:				**0.039**
Palliative Treatment Only	96 (49%)	72 (54%)	22 (38%)	
Non-Palliative Treatment	99 (51%)	61 (46%)	36 (62%)	
Surgery:				
Any Surgery	99 (50%)	60 (44%)	36 (62%)	**0.02**
Extrapleural Pneumonectomy	-	57 (42%)	-	
Extrapleural Decortication	-	3 (2.2%)	-	
Cytoreductive Surgery with HIPEC	-	-	30 (52%)	
Cytoreductive Surgery without HIPEC	-	-	6 (10%)	
Immunotherapy	102 (51%)	76 (55%)	26 (45%)	0.2
Neoadjuvant Chemotherapy	28 (14%)	16 (12%)	11 (19%)	0.2
Adjuvant Chemotherapy	46 (23%)	39 (28%)	7 (12%)	**0.014**
Palliative Chemotherapy	147 (74%)	107 (78%)	39 (67%)	0.11
Different Systemic Chemo Regimens Received:^b^				**0.005**
0	23 (12%)	8 (5.8%)	14 (25%)	
1	68 (35%)	52 (38%)	15 (26%)	
2	69 (35%)	48 (35%)	20 (35%)	
3	28 (14%)	20 (15%)	8 (14%)	
4	6 (3.0%)	6 (4.4%)	0 (0%)	
5	3 (1.5%)	3 (2.2%)	0 (0%)	
**Pathologic Findings**				
Histologic Subtype:				**<0.001**
Epithelioid	157 (80%)	99 (72%)	54 (96%)	
Biphasic	31 (16%)	30 (22%)	1 (1.8%)	
Sarcomatoid	9 (4.6%)	8 (5.8%)	1 (1.8%)	
Nuclear Grade:^c^				0.052
1	57 (38%)	29 (31%)	26 (51%)	
2	78 (52%)	55 (58%)	21 (41%)	
3	15 (10%)	11 (12%)	4 (7.8%)	
Nuclear Atypia Score:^c^				0.2
1	21 (14%)	10 (11%)	9 (18%)	
2	109 (74%)	68 (74%)	39 (76%)	
3	17 (12%)	14 (15%)	3 (5.9%)	
Mitotic Count Score:^c^				0.11
1	55 (37%)	29 (32%)	25 (49%)	
2	37 (25%)	24 (26%)	11 (22%)	
3	55 (37%)	39 (42%)	15 (29%)	
**Tumor Somatic Mutational Profiling**				
Tumor Mutational Burden, mean (SD)	2.01 (1.57)	2.05 (1.58)	1.88 (1.56)	0.4
Presence of Any Tumor Mutation	176 (88%)	123 (90%)	49 (84%)	0.3
Tumor Mutations:^d^				
*BAP1*	97 (49%)	65 (47%)	28 (48%)	>0.9
*CDKN2A*	51 (26%)	43 (31%)	8 (14%)	**0.011**
*NF2*	47 (24%)	34 (25%)	12 (21%)	0.5
*TP53*	42 (21%)	37 (27%)	5 (8.6%)	**0.004**
*TERT*	18 (9.0%)	13 (9.5%)	4 (6.9%)	0.6
*DDX3X*	12 (6.0%)	7 (5.1%)	5 (8.6%)	0.3
*PBRM1*	10 (5.0%)	3 (2.2%)	7 (12%)	**0.008**
*FBXW7*	6 (3.0%)	5 (3.6%)	0 (0%)	0.3
*CHEK2*	5 (2.5%)	4 (2.9%)	1 (1.7%)	>0.9
*PTEN*	5 (2.5%)	4 (2.9%)	1 (1.7%)	>0.9
*SETD2*	5 (2.5%)	5 (3.6%)	0 (0%)	0.3
*ATM*	4 (2.0%)	1 (0.7%)	3 (5.2%)	0.081
*NF1*	4 (2.0%)	2 (1.5%)	1 (1.7%)	>0.9
*ARID2*	3 (1.5%)	3 (2.2%)	0 (0%)	0.6
*MSH6*	3 (1.5%)	2 (1.5%)	1 (1.7%)	>0.9
*BRCA2*	2 (1.0%)	2 (1.5%)	0 (0%)	>0.9
*FAT3*	2 (1.0%)	1 (0.7%)	1 (1.7%)	0.5
*MDM2*	2 (1.0%)	2 (1.5%)	0 (0%)	>0.9
*NRAS*	2 (1.0%)	2 (1.5%)	0 (0%)	>0.9
*WT1*	2 (1.0%)	1 (0.7%)	1 (1.7%)	0.5

Overview of all patient characteristics including patient demographics, treatments, pathology, outcomes, and somatic mutation profiles with comparisons of characteristics for patients with pleural versus peritoneal mesothelioma.

*p*-value‡ comparing characteristics of patients with pleural mesothelioma versus peritoneal mesothelioma. Boldface entries indicate statistical significance (*p*-value < 0.05).

χ^2^test or Fisher’s exact test for categorical variables (number (%)) and t-test for continous variables (mean(standard deviation)).

^a^Includes four patients with bicavitary (both pleural and peritoneal) mesothelioma involvement.

^b^Number of Different Systemic Chemotherapy Regimens Received may include experimental regimens.

^c^Kadota System for scoring of variables and only includes tumors with epithelioid histologic subtype.

^d^Tumor Mutations shown for all mutations with ≥2 occurrences among the entire patient cohort. See Supplementary for the list of all gene mutations tested.

### Overall survival by treatment approach

**Fig. 1:**
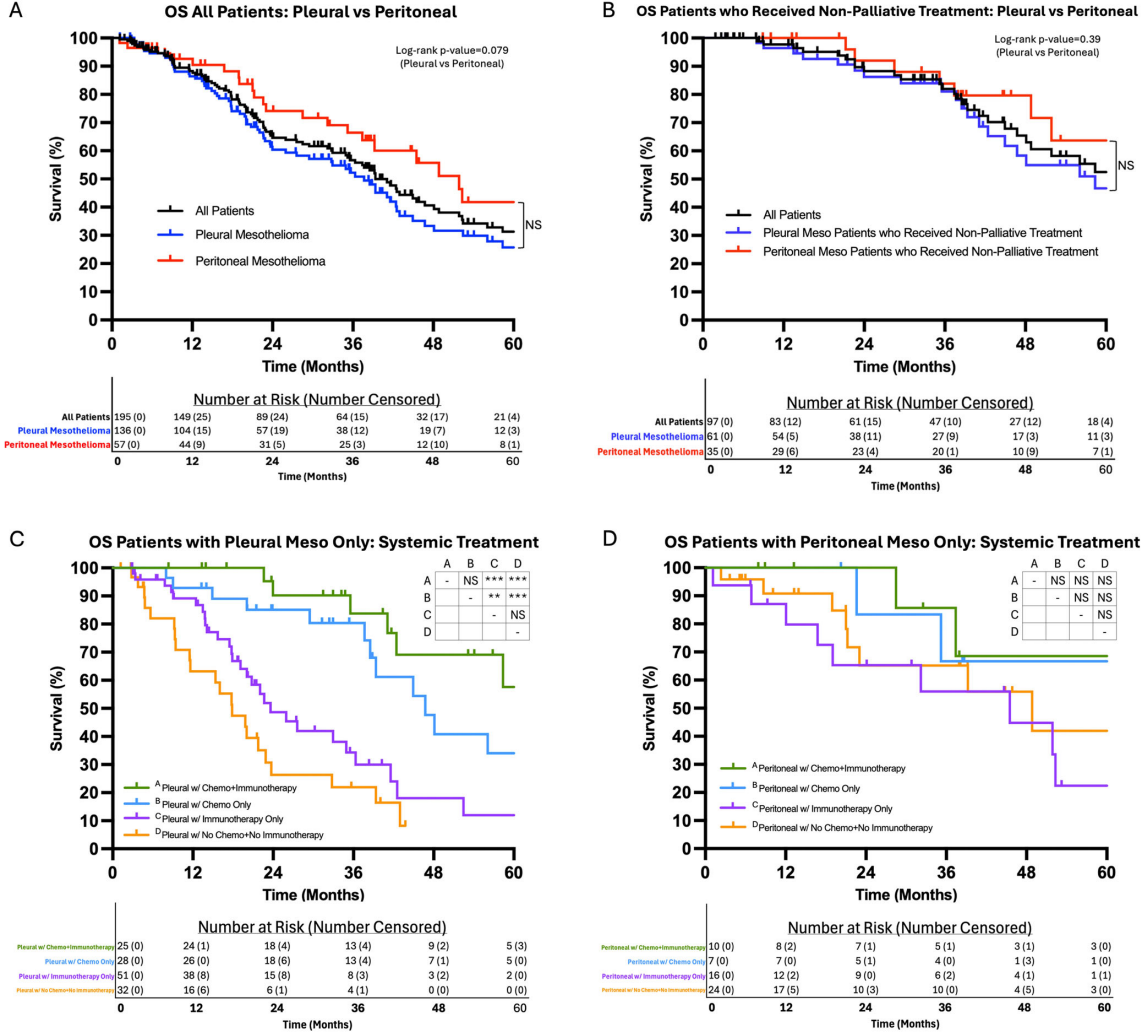
Overall survival (OS) from date of diagnosis for patients stratified by mesothelioma type and treatment. Overall survival (OS) of patients by diagnosis of pleural versus peritoneal mesothelioma was compared among (**A**) all patients in the cohort and (**B**) patients who received non-palliative treatment only. Survival analysis also evaluated (**C**) pleural mesothelioma patients only and (**D**) peritoneal mesothelioma patients only by the different combinations of systemic treatment received, including immunotherapy and/or any chemotherapy (neoadjuvant or adjuvant).

As expected, KM analysis showed worse OS in pleural compared to peritoneal mesothelioma although this was not statistically significant (log-rank *p* = 0.079) likely due to sample size. KM-estimated OS for peritoneal mesothelioma was 92.6% at 1-year, 74.1% at 2-years, and 41.8% at 5-years. Pleural mesothelioma estimated OS was 86.4% at 1-year, 60.4% at 2-years, and 25.7% at 5-years (Fig. 1A). Peritoneal mesothelioma patients who received non-palliative treatment had estimated OS of 100% at 1-year, 92.0% at 2-years, and 63.7% at 5-years whereas pleural mesothelioma patients had estimated OS of 96.5% at 1-year, 86.2% at 2-years, and 46.7% at 5-years (Fig. 1B).

Treatment with both immunotherapy and systemic chemotherapy (neoadjuvant and/or adjuvant chemotherapy) improved OS for pleural mesothelioma patients compared to patients who received only immunotherapy (log-rank p < 0.001) or who received no systemic treatment (log-rank p < 0.001) (Fig. 1C). Although similar trends for systemic treatment were seen in peritoneal mesothelioma, they were not significant (Fig. 1D).

### Tumor mutational profiling for pleural versus peritoneal mesothelioma

Among all patients, 88% (n = 176) had tumors with ≥1 somatic mutation. Among the 50-gene panel, the most common mutations included *BAP1* (49%, n = 97), *CDKN2A* (26%, n = 51), *NF2* (24%, n = 47), *TP53* (21%, n = 42), and *TERT* (9.0%, n = 18) (Table 1). The mean tumor mutational burden (TMB) among all patients was 1.77 mutations per patient with pleural mesothelioma having significantly higher TMB compared to peritoneal mesothelioma (1.90 vs 1.41, p = 0.0023). Pleural mesothelioma patients were more likely to have *CDKN2A* (31% vs 14%, p = 0.011) and *TP53* (27% vs 8.6%, p = 0.004) mutations but less likely to have *PBRM1* (2.2% vs 12%, p = 0.008) mutations compared to peritoneal mesothelioma patients (Table 1) (full list of gene panel in Supplementary Table 1).

**Fig. 2:**
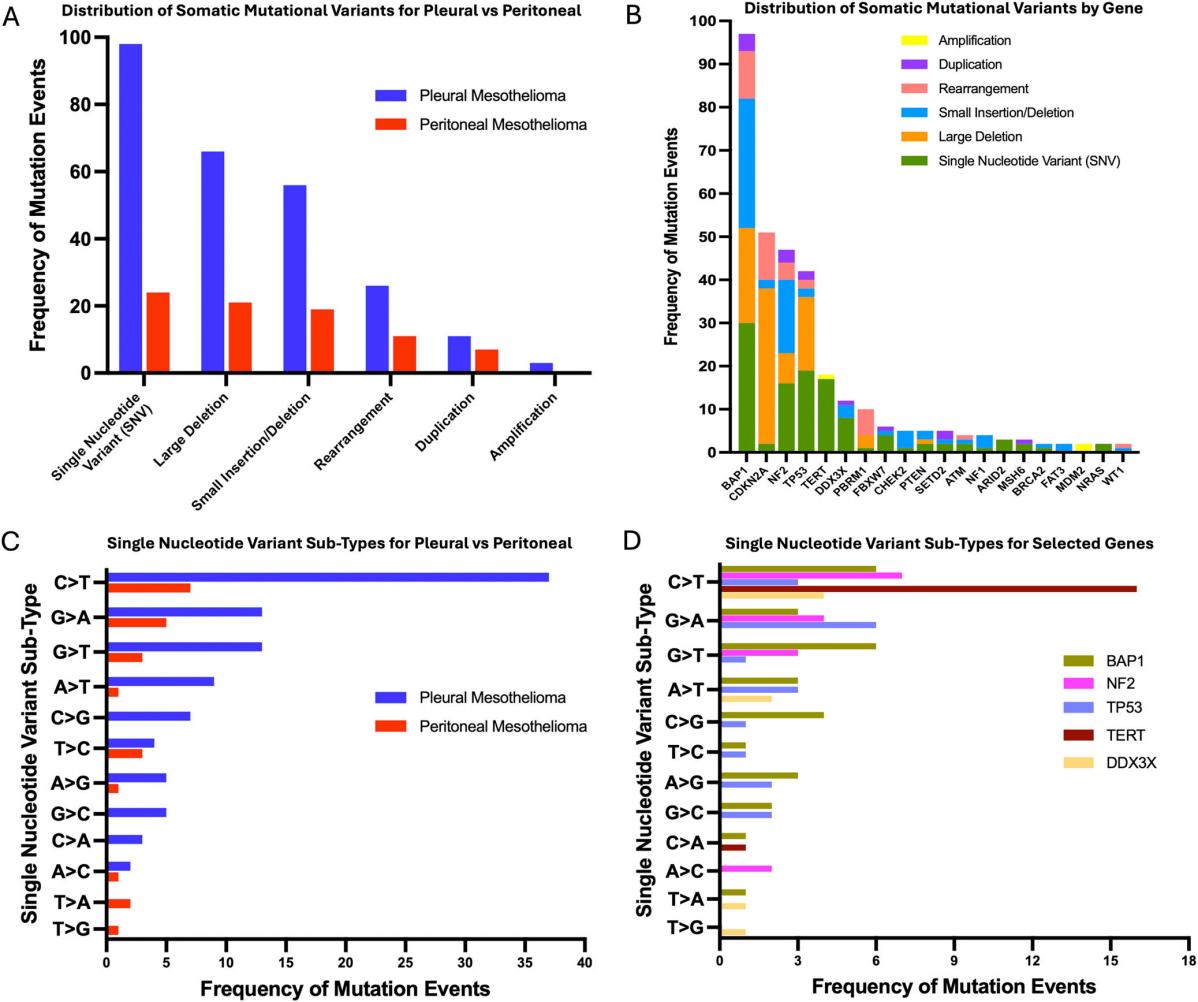
Distribution of somatic mutational variants and single nucleotide variant (SNV) sub-types for pleural and peritoneal mesothelioma. Next-generation sequencing of mesothelioma reveals patterns of somatic mutations among patients. **A** The frequency of mutational variant types is demonstrated by total number of mutations for pleural versus peritoneal mesothelioma patients. **B** The distribution of gene mutations was subdivided by variant type among all mesothelioma patients. **C** The SNV sub-type frequency was evaluated among pleural versus peritoneal mesothelioma patients. **D** SNV sub-type distribution was also determined for the genes with the highest frequency of SNV mutations among all mesothelioma patients.

The most common variants across all genes included single nucleotide variant (SNV) and large deletion whereas the least frequent variants included duplication and amplification (Fig. 2A). Among the most frequently mutated genes, the most common variant included 31% of *BAP1* mutants with SNV, 71% of *CDKN2A* mutants with large deletion, 36% of *NF2* mutants with small insertion/deletion (INDEL), 45% of *TP53* mutants with SNV, and 94% of *TERT* mutants with SNV (Fig. 2B). The most common SNV sub-types were C>T, G>A, and G>T with similar patterns seen in both pleural and peritoneal mesothelioma (Fig. 2C). SNV-subtypes among the genes with highest SNV frequency showed C>T transition in 94% of SNV *TERT* mutants and 40% of SNV *NF2* mutants (Fig. 2D).

### Tumor mutational profiling for mesothelioma histologic subtypes

Among all patients, 80% (n = 157) had epithelioid histologic subtype, including 72% (n = 99) of pleural and 96% (n = 54) of peritoneal mesothelioma patients. For tumors with epithelioid histologic subtype, pleural mesothelioma was significantly more likely to have *TP53* mutation (29% vs 9.3%, p = 0.004) and less likely to have *PBRM1* mutation (3.0% vs 13%, p = 0.034) compared to peritoneal mesothelioma (Supplementary Table 2). Pleural mesothelioma patients with epithelioid histologic subtype were significantly less likely to have *CDKN2A* (24% vs 50%, p = 0.004) or *TERT* (6.1% vs 18%, p = 0.046) mutations compared to patients with non-epithelioid (biphasic or sarcomatoid) histologic subtypes (Supplementary Table 3).

### Overall survival by key somatic mutations

**Fig. 3:**
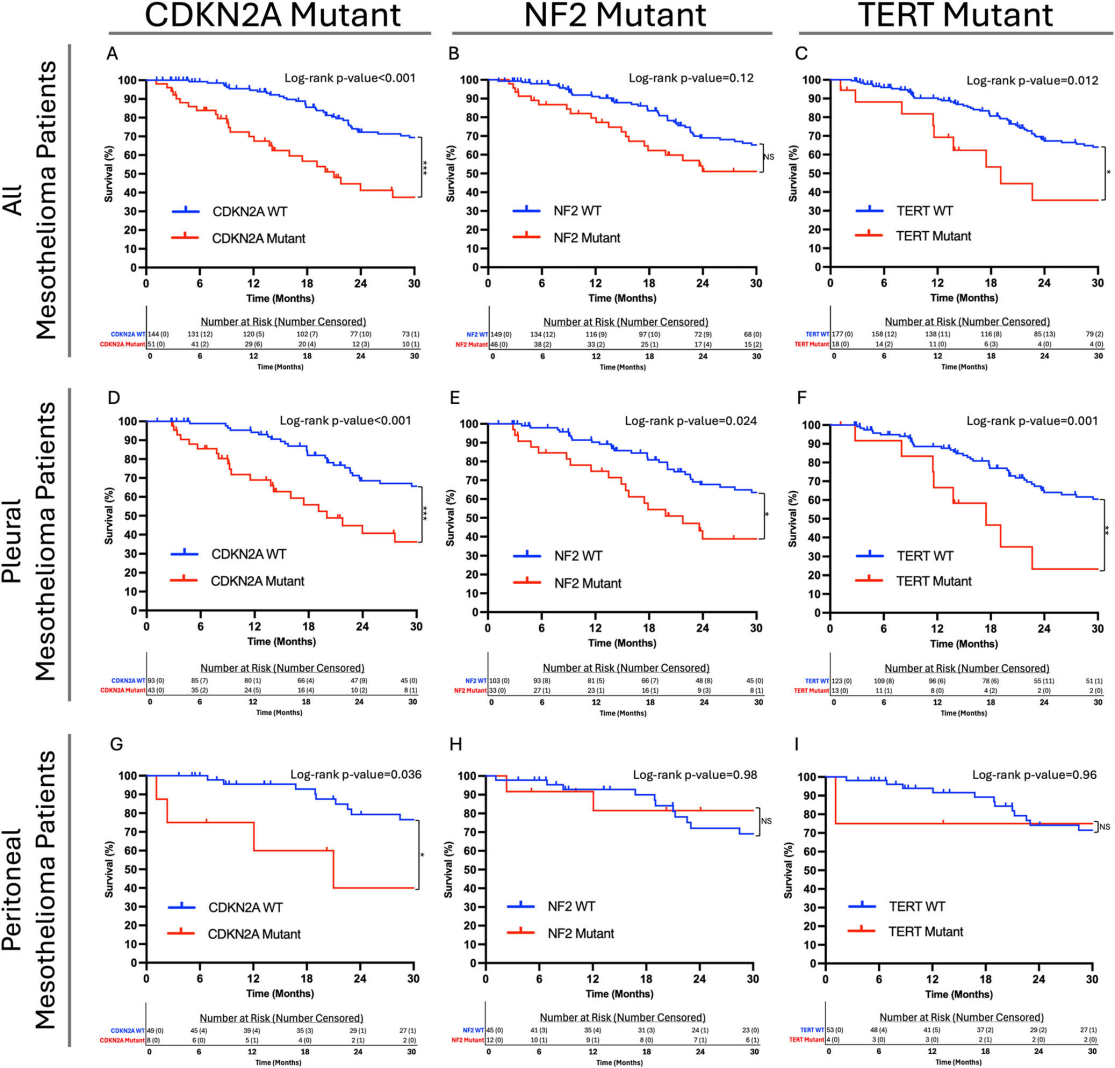
Overall survival from date of mesothelioma diagnosis for patients with selected somatic mutations with stratification by pleural and peritoneal mesothelioma. The overall survival (OS) was compared among all mesothelioma patients for the presence versus absence of **A** *CDKN2A* mutation, **B** *NF2* mutation, and **C** *TERT* mutation. Survival analysis was also conducted for only pleural mesothelioma patients by **D** *CDKN2A* mutation, **E** *NF2* mutation, and **F** *TERT* mutation as well as for only peritoneal mesothelioma patients by **G** *CDKN2A* mutation, **H** *NF2* mutation, and **I** *TERT* mutation.

Patients with *CDKN2A* mutations had significantly worse OS in both pleural (log-rank p < 0.001) (Fig. 3D) and peritoneal (log-rank p = 0.036) (Fig. 3G) mesothelioma. This included patients with *CDKN2A* mutations in the pleural mesothelioma group having lower 1-year OS (60.0% vs 93.1%), 2-year OS (33.3% vs 65.3%), and 5-year OS (0.0% vs 31.0%) as well as patients in the peritoneal mesothelioma group having lower 1-year OS (66.7% vs 94.6%), 2-year OS (33.3% vs 78.4%), and 5-year OS (0.0% vs 42.1%) compared to *CDKN2A* wildtype. Similar trends from KM analysis were seen for pleural mesothelioma patients with *NF2* mutations (log-rank p = 0.024) including lower 1-year OS (74.8% vs 90.3%), 2-year OS (38.8% vs 67.8%), and 5-year OS (19.4% vs 28.2%) compared to *NF2* wildtype (Fig. 3E). KM analysis for pleural mesothelioma patients with *TERT* mutations also had worse OS (log-rank p = 0.001) including lower 1-year OS (66.7% vs 88.5%), 2-year OS (23.3% vs 64.0%), and 5-year OS (0.0% vs 29.1%) compared to *TERT* wildtype (Fig. 3F). *BAP1* did not show significant survival differences, however, this may have been limited by sample size (Supplementary Fig. 1). Unlike patients with pleural mesothelioma, patients with peritoneal mesothelioma did not show significant differences in OS for either *NF2* (Fig. 3H) or *TERT* (Fig. 3I) mutations.

A similar KM analysis was conducted for all pleural mesothelioma patients to compare OS between epithelioid and non-epithelioid histologic subtypes. Pleural mesothelioma patients with epithelioid histologic subtype had significantly improved OS compared to sarcomatoid subtype (log-rank p = 0.013) (Supplementary Fig. 2). Furthermore, pleural mesothelioma patients with epithelioid histologic subtype had significantly worse OS if they had *CDKN2A* mutation (log-rank p = 0.0016) compared to wildtype (Supplementary Fig. 3). In contrast, pleural mesothelioma patients with non-epithelioid histologic subtypes (biphasic or sarcomatoid) had significantly worse OS if they had mutations in *NF2* (log-rank p = 0.040) or *TERT* (log-rank p = 0.011).

### Independent association of survival by somatic mutations

Multivariable Cox proportional hazards regression evaluated the independent association of mutations and clinical factors on survival. Significant prognostic factors identified on univariate analysis were included in the multivariate model—patient age, sex, tumor location, histology, systemic treatment, and significant mutations. Among all patients, mutations in *NF2* (HR [95% CI] = 1.74 [1.08–2.81], p = 0.024) and *TERT* (HR [95%CI] = 2.28 [1.16–4.50], p = 0.017) were independently associated with worse OS (Table 2). Similarly, pleural mesothelioma patients also showed an independent association with worse OS for mutations in *NF2* (HR [95%CI] = 1.95 [1.14–3.34], p = 0.015) and *TERT* (HR [95%CI] = 2.51 [1.16–5.44], *p* = 0.020). Analysis of peritoneal mesothelioma patients did not reveal any significant independent associations (Table 2).

**Table 2:** Multivariable Cox proportional hazards regression for survival

	All Mesothelioma Patients		Pleural Mesothelioma Patients		Peritoneal Mesothelioma Patients	
Variable	Adjusted Hazard Ratio (95% CI)	p-value	Adjusted Hazard Ratio (95% CI)	p-value	Adjusted Hazard Ratio (95% CI)	p-value
Female Sex	0.74 (0.45–1.19)	0.21	0.77 (0.44–1.37)	0.38	0.70 (0.27–1.81)	0.46
Older Age at Diagnosis (per year)	1.02 (1.00–1.04)	0.052	1.02 (0.99–1.05)	0.22	1.02 (0.99–1.06)	0.24
Peritoneal Mesothelioma	0.71 (0.42–1.19)	0.19	-	-	-	-
Epithelioid Histologic Subtype	0.78 (0.44-1.37)	0.38	0.78 (0.43–1.42)	0.42	0.58 (0.05–6.67)	0.66
Received Neoadjuvant or Adjuvant Chemo	0.31 (0.20–0.51)	**<0.001**	0.28 (0.16–0.48)	**<0.001**	0.56 (0.18–1.76)	0.32
*CDKN2A* Mutation	1.50 (0.91–2.47)	0.11	1.36 (0.76–2.43)	0.29	2.42 (0.78–7.49)	0.12
*NF2* Mutation	1.74 (1.08–2.81)	**0.024**	1.95 (1.14–3.34)	**0.015**	0.91 (0.25–3.37)	0.89
*TERT* Mutation	2.28 (1.16–4.50)	**0.017**	2.51 (1.16–5.44)	**0.020**	2.24 (0.31–15.95)	0.42

All clinically relevant variables from Table 1 were compared individually with survival on univariate Cox regression. Variables with *p*-value ≤0.20 on univariate Cox regression underwent multivariable Cox regression with calculation of adjusted hazard ratios and corresponding *p*-values. Multivariable Cox regression was then repeated separately for pleural and peritoneal mesothelioma cohorts as shown in Table 2.

### Co-occurrence of mutations

**Fig. 4:**
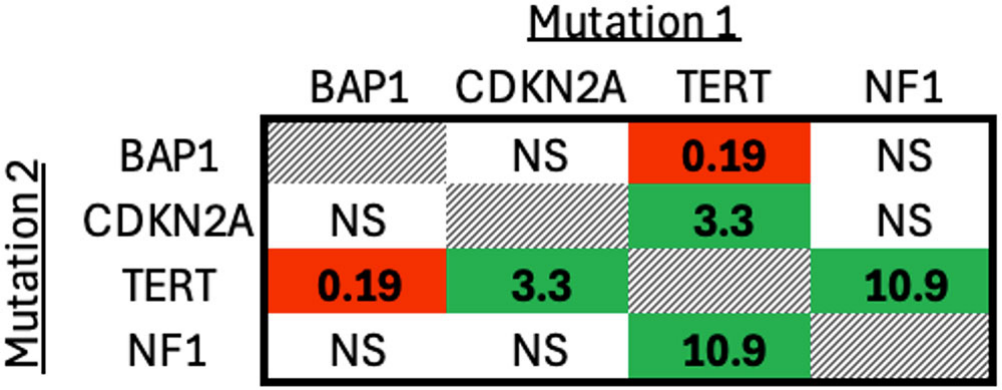
Pairwise odds ratios for co-occurrence of somatic mutations. All gene mutations from Table 1 and Supplementary Table 1 underwent pairwise odds ratio analysis with Fisher’s Exact Test. The pairwise genes with significant *p*-value < 0.05 are shown including *BAP1*, *CDKN2A*, *TERT*, and *NF1*. The values in the table are the pairwise odds ratios for corresponding pairs of gene mutations where values < 1.0 represent negative co-occurrence (red boxes) and values > 1.0 represent positive co-occurrence (green boxes) with higher values indicating higher likelihood of co-occurrence among corresponding gene mutations. Boxes with non-significant p-values < 0.05 on pairwise analysis are represented by the label NS.

Fisher’s Exact Test identified significant co-occurrence patterns among somatic mutations. *TERT* mutation had a 3.3× higher risk of co-occurrence with *CDKN2A* (OR [95%CI] = 3.3 [1.08–10.0], p = 0.02) and 5.3× lower risk of co-occurrence with *BAP1* (OR [95%CI] = 0.19 [0.03–0.69], p = 0.004) (Fig. 4). No significant mutational co-occurrences were seen for *NF2*.

### Mutational profiles for patients with shortened versus extended survival

**Fig. 5:**
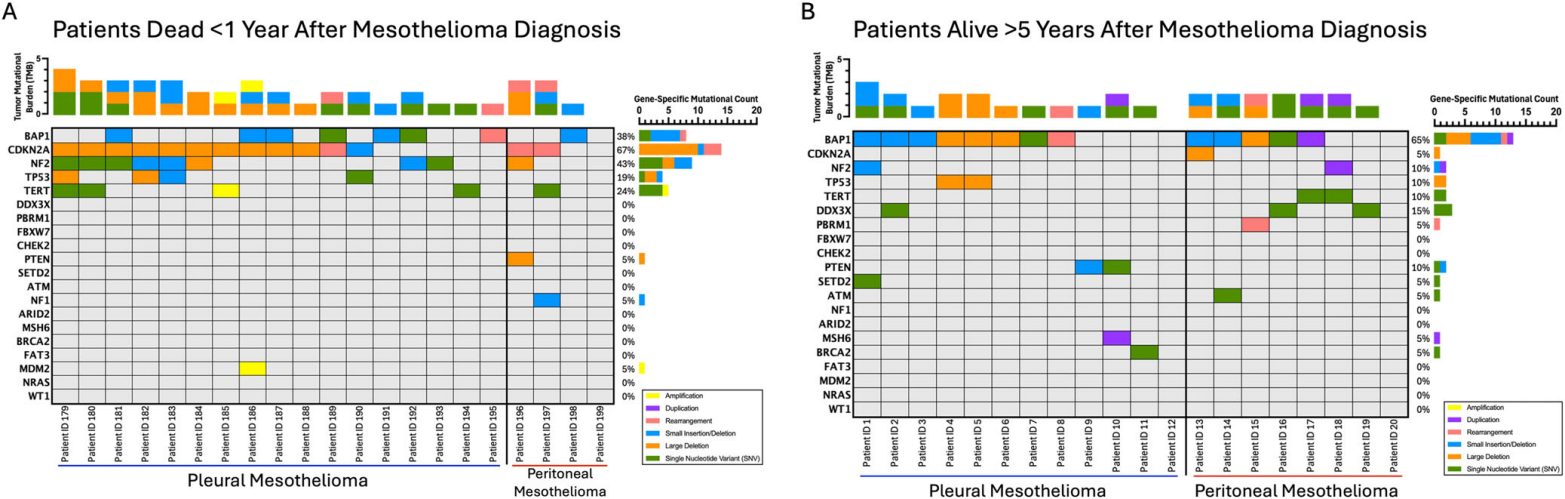
Oncoplots of somatic mutations for patients with <1 year survival and >5 years survival from date of diagnosis. Oncoplots demonstrate the somatic mutational profiles of (**A**) all patients with <1 year overall survival and (**B**) patients with >5 years overall survival. Oncoplots demonstrate the corresponding gene mutations captured for the top 20 most frequently mutated genes. Mutational variants of each gene are shown with color coding, where yellow=amplification, purple=duplication, pink=rearrangement, blue=small insertion or small deletion, orange=large deletion, and green=single nucleotide variant.

Oncoplots compared patients with shortened survival (<1-year OS) (Fig. 5A) or extended survival (>5-year OS) (Fig. 5B). Among the 21 patients with <1-year OS, the most common mutation was *CDKN2A* (67%, n = 14), with 71% (n = 10) of *CDKN2A* mutants being a large deletion. Patients with <1-year OS also had high frequency of *NF2* (43%, n = 9) and *TERT* (24%, n = 5) mutations with 80% (n = 4) of *TERT* mutations co-occurring with *CDKN2A* mutation (Fig. 5A).

Compared to patients with >5-year OS, patients with <1-year OS had significantly higher mean tumor mutational burden (TMB) (2.14 vs 1.50, p = 0.019). Patients with <1-year OS also had significantly higher frequency of *CDKN2A* (67% vs 5.0%, p < 0.001) and *NF2* (43% vs 10%, p = 0.018) mutations compared to patients with >5-year OS (Supplementary Table 4). Furthermore, among pleural mesothelioma patients with <1-year OS, *NF2* was the 2nd most common mutation with 50% (4/8) SNV, 38% (3/8) small deletion, and 13% (1/8) large deletion. Of the SNV mutants, 100% (4/4) were from C>T transition and resulted in a non-sense mutation. Similarly, 100% (3/3) of small deletion mutants resulted in frameshift mutations with premature stop codon (Fig. 5A). Among all pleural mesothelioma patients with SNV mutations in *NF2*, 100% (6/6) of patients with C>T transition died within 16-months of diagnosis which was significantly worse than patients with non-C>T transitions in *NF2* (17% mortality in 16-months, p = 0.015). All patients with C>T transitions in *NF2* were non-sense mutations; and although C> T transition in *NF2* was seen in 50% (6/12) of all pleural mesothelioma patients with SNV, 0% of peritoneal mesothelioma patients had C>T transition in *NF2*.

Among all patients with <1-year OS, *TERT* was the 4th most common gene mutation with 80% of *TERT* mutations being SNV and 100% being C>T transition. Unlike *NF2* where the rate of C>T transition among the entire cohort was relatively low (50% in pleural mesothelioma), the rate of C>T transition in patients with SNV for *TERT* was 100% (16/16) and comprised 94% (16/17) of all *TERT* mutations. Additionally, among all patients with *TERT* mutation, 46% (6/13) died at 16 months, and 69% (9/13) died at 2-years after diagnosis—both of which are significantly higher than the mortality rates of patients without *TERT* mutations (16% mortality at 16-months, p = 0.018; and 36% mortality at 2-years, p = 0.021).

### Survival impact of *NF2* truncating variants

**Fig. 6:**
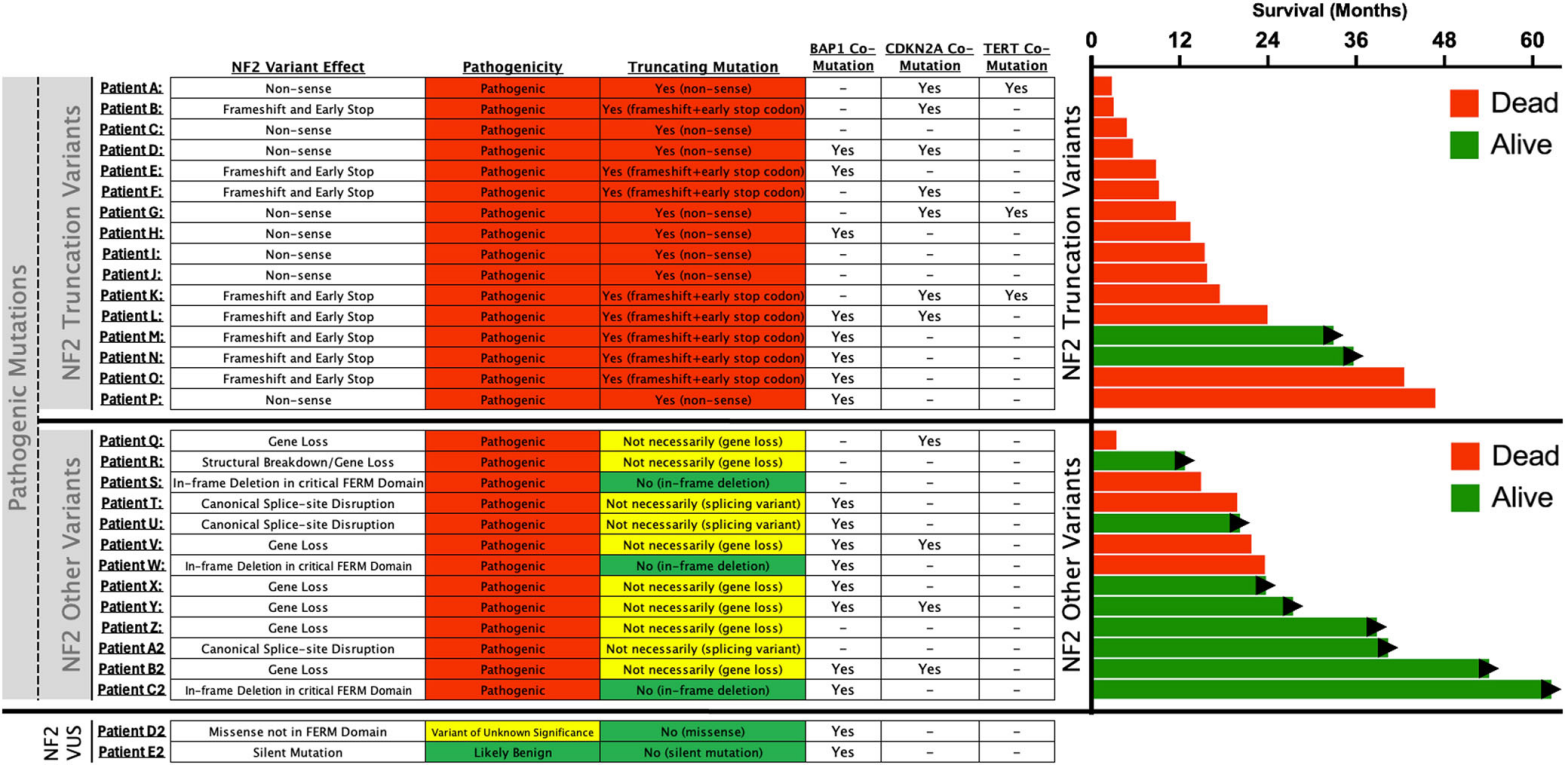
Hybrid oncoplot of pathogenic *NF2*-mutant pleural mesothelioma showing overall survival and *NF2*-variant transcription and translational effects. All patients with pleural mesothelioma and *NF2* mutation are shown in the figure as individual patients with detailed information on the specific pathogenic variants^34,48^ (full mutational details from pathology report shown for all patients in Supplementary Fig. 4), select co-mutations, determination of truncating versus non-truncating mutation, and overall survival from date of diagnosis. Truncating mutations (Patient A – Patient P) are defined as mutations which cause production of a truncated NF2 protein, specifically, a non-sense mutation or a frameshift mutation with introduction of a premature stop codon. The bar chart shows the survival of the patients in months with red bars for deceased patients (date of diagnosis to date of death) and green bars for alive patients (date of diagnosis to date last seen).

Pleural mesothelioma patients who died within 1-year commonly had *NF2* mutation (Fig. 5A). Of patients with *NF2* mutation, 88% (7/8) had a truncating mutation in *NF2,* including 50% (4/8) with SNV C>T transition and 38% (3/8) with small deletions, which all resulted in a premature stop codon. To further investigate the prognostic potential of *NF2* mutations with protein-truncating variants, all pleural mesothelioma patients with a pathogenic *NF2* mutation who either died or had >1-year follow-up were stratified as *NF2* truncating variants (55%, n = 16) or other *NF2* pathogenic variants (45%, n = 13) (Fig. 6) (see Supplementary Fig. 4 for the detailed pathologic mutation report of each patient). Patients with non-pathogenic *NF2* mutations including variants of unknown significance (n = 1) or silent mutations (n = 1) were excluded. Patients with *NF2* truncating variants had significantly higher mortality at 1-year (44% vs 7.7%, p = 0.044) and 18-months (69% vs 17%, p = 0.006) compared to patients with other *NF2* pathogenic variants.

**Fig. 7:**
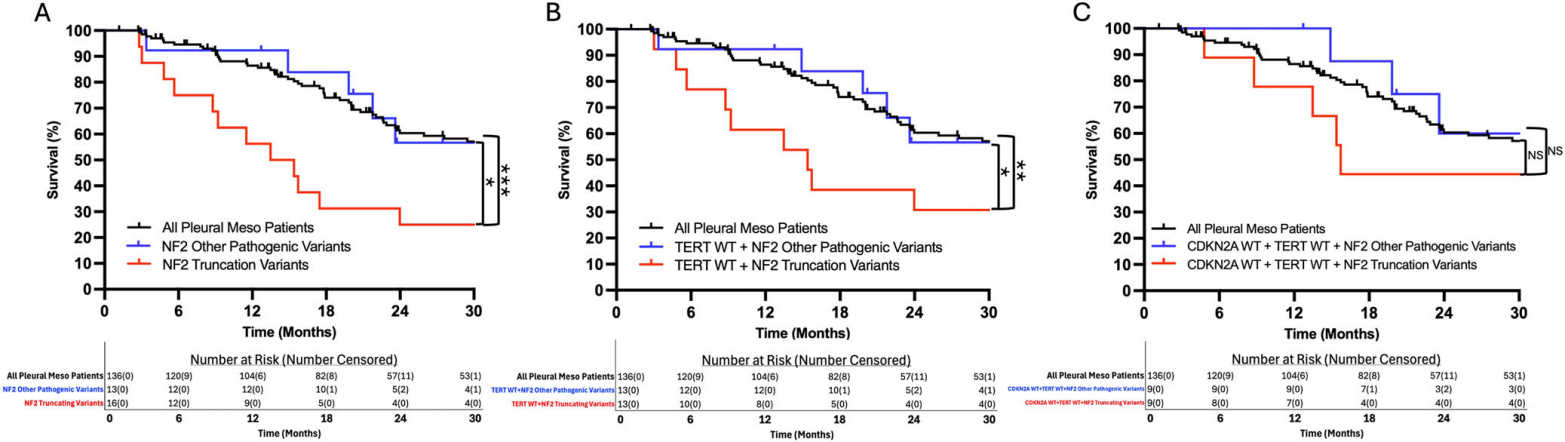
Overall survival from date of diagnosis for pleural mesothelioma patients by *NF2* mutational variants. Overall survival (OS) was investigated for (**A**) *NF2*-mutant pleural mesothelioma patients by the presence of *NF2* truncating mutations versus the presence of other pathogenic non-truncating *NF2* mutations. Survival analysis was also conducted for the same patient cohort except with exclusion of patients with either (**B**) *TERT* mutation (represented by inclusion of only *TERT* wildtype [WT] patients) or **C** *TERT* and/or *CDKN2A* mutations (represented by inclusion of only *TERT* and *CDKN2A* WT patients). The black line for each graph represents OS for all patients with pleural mesothelioma regardless of somatic mutation profile. The red line represents pleural mesothelioma patients with *NF2* truncating mutations, including non-sense mutations and frameshift mutations which introduce a premature stop codon. The blue line represents pleural mesothelioma patients with *NF2* mutations which are not truncating variants—these can include variants such as large chromosomal deletions or rearrangements, missense mutations, and silent mutations (see Fig. 6 for more details of patient distribution and characteristics of *NF2* variants).

KM analysis confirmed these findings with significantly worse OS in pleural mesothelioma patients with *NF2* truncating variants compared to patients with other *NF2* pathogenic variants (log-rank p = 0.013) (Fig. 7A). Interestingly, there was no difference in OS for patients with pathogenic non-truncating *NF2* variants compared to all pleural mesothelioma patients, suggesting that the poor prognostic influence of *NF2* mutations may come from the *NF2* truncating variants specifically. Of note, *NF2* truncating variants were not associated with the co-occurrence of BAP1, CDKN2A, TERT, or other genes. Repeat KM analysis for the cohort after exclusion of TERT mutants still showed significantly worse OS for *NF2* truncating variants (log-rank p = 0.038) compared to other *NF2* pathogenic variants (Fig. 7B). Similar trends are seen after exclusion of both TERT and CDKN2A mutants (Fig. 7C), however, this was not significant due to sample size.

Multivariable Cox proportional hazards regression showed that among pleural mesothelioma patients, *NF2* truncating mutations were independently associated with worse OS (HR [95%CI] = 3.32 [1.71–6.43], p < 0.001) regardless of sex, age, histology, systemic chemotherapy, or presence of *CDKN2A* or *TERT* mutations (Table 3). Importantly, when *NF2* pathogenic mutations are characterized as non-truncating variants, there is no significant effect on OS (HR [95%CI] = 0.91 [0.35–2.35], p = 0.84), suggesting again that the negative prognostic impact of *NF2* mutations is likely heavily influenced by truncating variants specifically. However, additional studies need to be conducted to validate these findings and to explore the mechanism through protein-level evaluation.

**Table 3:** Multivariable Cox proportional hazards regression for survival of pleural mesothelioma patients

	Pleural Mesothelioma Patients	
Variable	Adjusted Hazard Ratio (95% CI)	p-value
Female Sex	0.66 (0.37–1.19)	0.17
Older Age at Diagnosis (per year)	1.02 (0.99–1.05)	0.13
Epithelioid Histologic Subtype	0.87 (0.49–1.57)	0.65
Received Neoadjuvant or Adjuvant Chemo	0.31 (0.18–0.54)	**<0.001**
***NF2*** **Truncation Mutation**	3.32 (1.71–6.43)	**<0.001**
***NF2*** **Non-Truncation Pathogenic Mutation**	0.91 (0.35-2.35)	0.84
*CDKN2A* Mutation	1.50 (0.83–2.73)	0.18
*TERT* Mutation	1.91 (0.84-4.35)	0.12

Using the same backward elimination technique from Table 2, clinically relevant variables and key mutations were compared to determine independent association with OS. In contrast to prior methods of using *NF2* mutation as one variable, *NF2* mutation was divided into *NF2* Truncation Mutation or *NF2* Non-Truncation Pathogenic Mutation using the tumor profiling details shown in Fig. 6.

## Discussion

In this large cohort of patients with mesothelioma, we demonstrate that pleural and peritoneal mesothelioma subtypes have unique somatic mutational profiles which carry prognostic significance. In addition to conducting large-scale genomic and survival analysis, we uncovered the independent prognostic association of two key mutations—*TERT* and *NF2* truncating mutations—for patients with pleural mesothelioma. Overall, these findings reiterate the need to obtain somatic testing for patients with mesothelioma to improve prognostication, guide clinical-trial enrollment, and ultimately, to develop targeted-treatment approaches.

Although the prognostication of *NF2* in pleural mesothelioma is moderately well characterized from prior studies^21,24^, we identified on the molecular level that the poor survival associated with *NF2* is likely due to specific variants resulting in protein truncation. Interestingly, there are many clinical trials which are therapeutically targeting factors in the *NF2* pathway. For example, the VT3989 phase 1/2 clinical trial is targeting TEAD, a downstream transcription factor in the *NF2* pathway, and received FDA fast track designation in October 2025^27,28^. Prior clinical trials such as the COMMAND phase 2 trial failed to show survival benefit in pleural mesothelioma patients receiving targeted inhibition of FAK protein, another downstream pathway from *NF2*^29^. Our work shows that, specifically, the truncation of NF2 mutant protein may play a significant role compared to other *NF2* pathogenic variants. Importantly, however, this finding should be further explored with protein-level evaluation and validation in prospective, multi-institutional studies. Using genomic level information about *NF2* variants could help identify appropriate patients for clinical trials or encourage new therapeutic targets for pleural mesothelioma patients with these genomic signatures.

Our study also identified other frequent somatic alterations, including *BAP1*, *CDKN2A*, and *TP53*, mirroring prior reports^18,30^. However, we also showed that *TERT* mutations are independently associated with worse overall survival in pleural mesothelioma patients, which was previously demonstrated in survival studies by a few, small European studies^25,26^. As there are currently no clinical trials evaluating *TERT*-specific therapeutic targets, the validation of *TERT* as a negative prognostic factor deserves further investigation.

On univariate survival analysis, our study showed *CDKN2A* mutation was a poor prognostic marker for both pleural and peritoneal mesothelioma; however, we found that this was not significant on multivariate analysis. Although the results of other studies are mixed, most studies agree that there is a likely association of *CDKN2A* mutation with poor prognosis in mesothelioma^24,30–33^. However, our study did not fully support this trend. Instead, we found that although *CDKN2A* mutation may play a role, the major factor accounting for poor survival in these patients might be *TERT* mutation – which was independently associated with worse OS and had a 3.3x higher chance of co-occurring with *CDKN2A* mutation. Prior studies of pleural mesothelioma patients with *BAP1* somatic mutations have shown mixed prognostic results with most studies demonstrating somatic *BAP1* mutations are not predictive of survival, similar to our results^34,35^. Importantly, germline *BAP1* mutations are widely shown to be associated with improved survival although our study did not have germline data available for analysis^35^. Regardless, these mutational patterns are highly complex and emphasize the utility of integrating genomic data into baseline risk stratification, especially for pleural mesothelioma patients^36,37^. In contrast, our study did not find any independent prognostic impact of somatic mutations in peritoneal mesothelioma patients. This divergence suggests pleural mesothelioma outcomes may be more genomically-driven, while surgical and treatment variables may dominate prognosis in peritoneal disease^38,39^.

This study has several limitations which should be acknowledged. First, this study uses a retrospective design conducted within a single health system, which may introduce the potential for selection bias and reduce the study’s generalizability to broader populations. Although we minimized this bias with the inclusion of all sequential patients with NGS testing during 2010–2022, patients who did not receive NGS testing were excluded. Second, although this cohort represents one of the largest cohorts of mesothelioma patients with integrated genomic and survival data, the sample size within specific mutational subgroups remains relatively modest and may limit statical power. Third, although patients underwent NGS testing with the same gene panel and the same institutional tissue processing protocols, there is operator bias in how these protocols are conducted which may affect mutational detection sensitivity in some cases. Fourth, immunohistochemical protein-level validation and germline genetic testing were not available for this study. Absence of these prevent the confirmation of mutant-corresponding protein expression changes and evaluation of potential hereditary predisposition, respectively. Specifically, germline *BAP1* mutations are associated with improved survival and are more common in female mesothelioma patients^35^. Our study cohort notably included 62% female which may have presented higher rates of germline *BAP1* mutations. Although patient sex was accounted for in the multivariate analysis, the inclusion of germline mutation data would have decreased confounding effects. Lastly, survival analysis may remain subject to additional residual confounding factors despite the multivariable adjustments that we made. As such, prognostic findings warrant further validation in larger prospective cohorts.

Our findings support the integration of somatic mutation testing into clinical prognostication, particularly in pleural mesothelioma. For peritoneal disease, treatment decisions should remain guided by clinical factors such as histology, tumor burden, and resectability, while the prognostic value of genomic alterations warrants further study^21,40–43^. More broadly, our data emphasize that pleural and peritoneal mesothelioma are biologically distinct with unique somatic mutational profiles and should not be extrapolated across one another in clinical trial design^44,45^. Key somatic mutations in pleural mesothelioma, including *TERT* mutations and *NF2* truncating mutations, should be further explored with prospective outcomes in multicenter studies. Ultimately, our findings highlight the mutational heterogeneity of mesothelioma and support site-specific, biomarker-informed strategies to guide trial enrollment and to aid in the advancement of targeted therapies for mesothelioma.

## Methods

### Study design and patient selection

All sequential patients ≥18-years-old who had somatic mutation profiling and confirmed diagnosis of pleural or peritoneal mesothelioma between 2010 and 2022 at the University of Chicago Mesothelioma Clinic were enrolled in an IRB-approved biorepository and database protocol (IRB #15-0443), conducted in accordance with the Declaration of Helsinki. All individuals in this study provided informed consent and voluntary participation in this study, including consent to research use of clinical and molecular data. All patients who consented and met the inclusion criteria were included in the study regardless of survival outcomes or specific genomic findings. Patients without somatic mutational profiling or with diagnosis of benign multicystic mesothelioma or well-differentiated papillary mesothelial tumor were excluded. Subsequent analysis performed at Yale used only fully deidentified data transferred from University of Chicago. No Yale patient data were included, and this secondary analysis of data was confirmed to not require Yale IRB review.

### Tissue collection and next-generation sequencing

Mesothelioma tissue samples were obtained in the form of formalin-fixed paraffin-embedded (FFPE) specimens during surgical resection or image-guided biopsy. A representative FFPE was selected for next-generation sequencing (NGS) on the University of Chicago Medicine-OncoPlus panel, a hybrid-capture panel targeting 1005 cancer-associated genes, of which 168 genes are clinically reported. DNA extraction, quantification, library preparation, NGS, and analysis were performed as described previously with standard, validated institutional protocols^46^.

### Chart review and patient characteristics

Retrospective chart review collected patient demographics, including age, sex, race, and clinical and pathologic characteristics such as primary mesothelioma site, stage, asbestos exposure, and histologic subtype. Treatment history was collected, including surgery and systemic therapy regimens. Patient characteristics were compared, and Kaplan-Meier (KM) analysis evaluated overall survival (OS) among patient groups.

### Somatic mutation profiles and survival impact

Tumor mutation profiles underwent detailed analysis to capture the distribution of mutational variants, gene-specific data, and single-nucleotide variant (SNV) subtypes. KM analysis identified mutations associated with worse OS. Pairwise odds ratio analysis with Fisher’s Exact Test captured co-occurrence frequencies of mutations. Each gene underwent pairwise comparisons with all other genes to identify significant co-occurrences of gene pairs.

Mutational data and clinical variables were first evaluated using univariate analysis to identify clinically relevant, significant candidate variables, which were subsequently used in multivariable Cox proportional hazards regression models. Additional analysis was conducted on significant mutations, including *TERT* and *NF2*^47,48^, to better characterize specific variants and their prognostic impact. Oncoplots were created to compare mutational patterns and tumor characteristics among patients with shortened survival (<1-year OS) and patients with extended survival (>5-year OS). All statistical analyses were conducted with R Studio (RRID:SCR_000432).

## Supplementary information

Supplementary Information

## Data Availability

The datasets generated and/or analyzed during the current study are not publicly available due to institutional review board (IRB) and patient privacy restrictions of our prospectively maintained institutional registry, but de-identified data may be made available from the corresponding author on reasonable request. The analytical code used for this study is available from the corresponding author on reasonable request.

## Code availability

The analytical code used for this study is available from the corresponding author on reasonable request.
