# Supplementary material for: Prognostic impact of somatic mutations among patients with pleural and peritoneal mesothelioma
Source: NPJ Precis Oncol. 2026 Jul 9;10:281. doi: 10.1038/s41698-026-01495-x (PMC13373184; doi:10.1038/s41698-026-01495-x)
Supplement: Supplementary file 1 — Supplementary Information [file 41698_2026_1495_MOESM1_ESM.pdf]

Supplemental Figures:

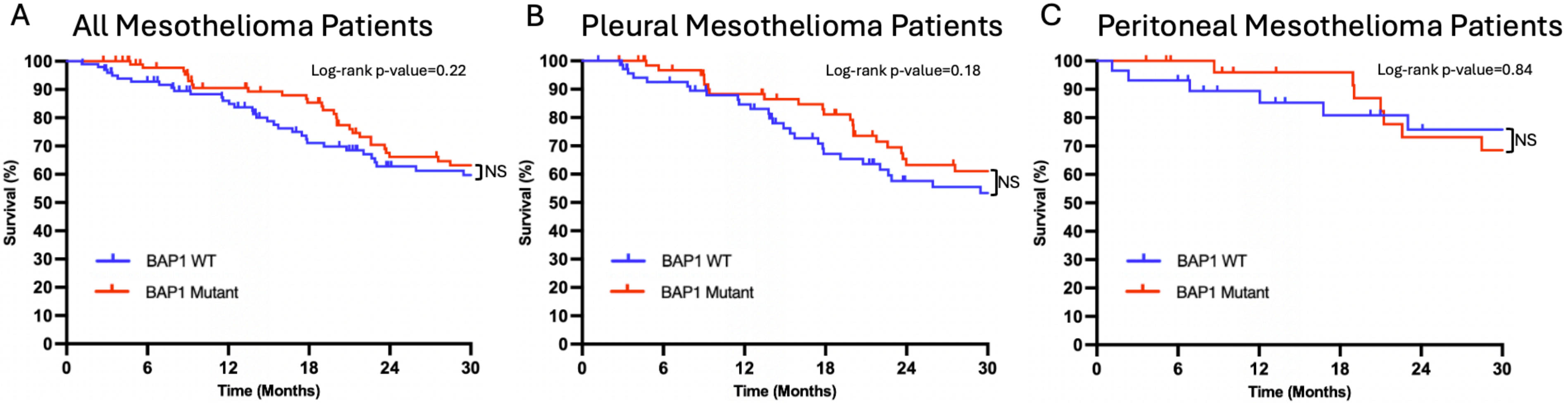

<u>Supplemental Figure 1:</u> <u>Overall Survival for Mesothelioma Patients with *BAP1* Somatic Mutation.</u> The overall survival (OS) was compared for mesothelioma patients with *BAP1* somatic mutations including among **(a)** all patients, **(b)** pleural mesothelioma patients only, and **(c)** peritoneal mesothelioma patients only.

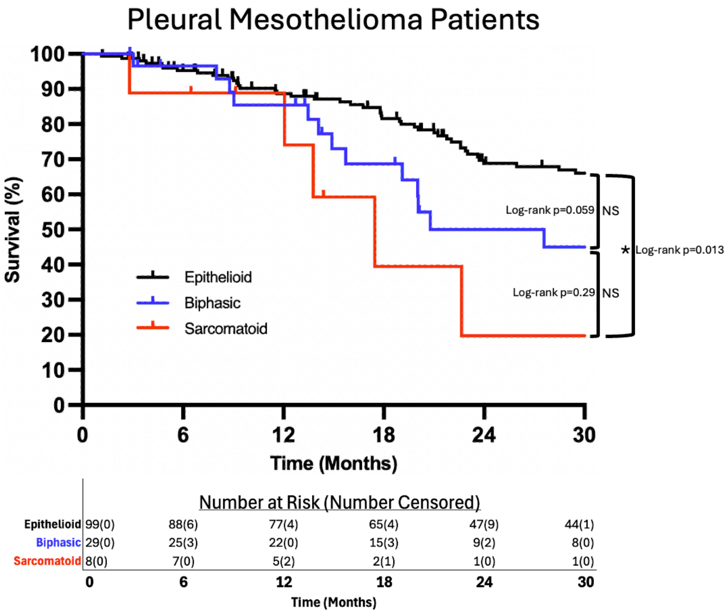

<u>Supplemental Figure 2:</u> <u>Overall Survival for Pleural Mesothelioma Patients by Histologic Subtype.</u> Overall survival (OS) was compared for pleural mesothelioma patients by histologic subtypes including epithelioid, biphasic, and sarcomatoid.

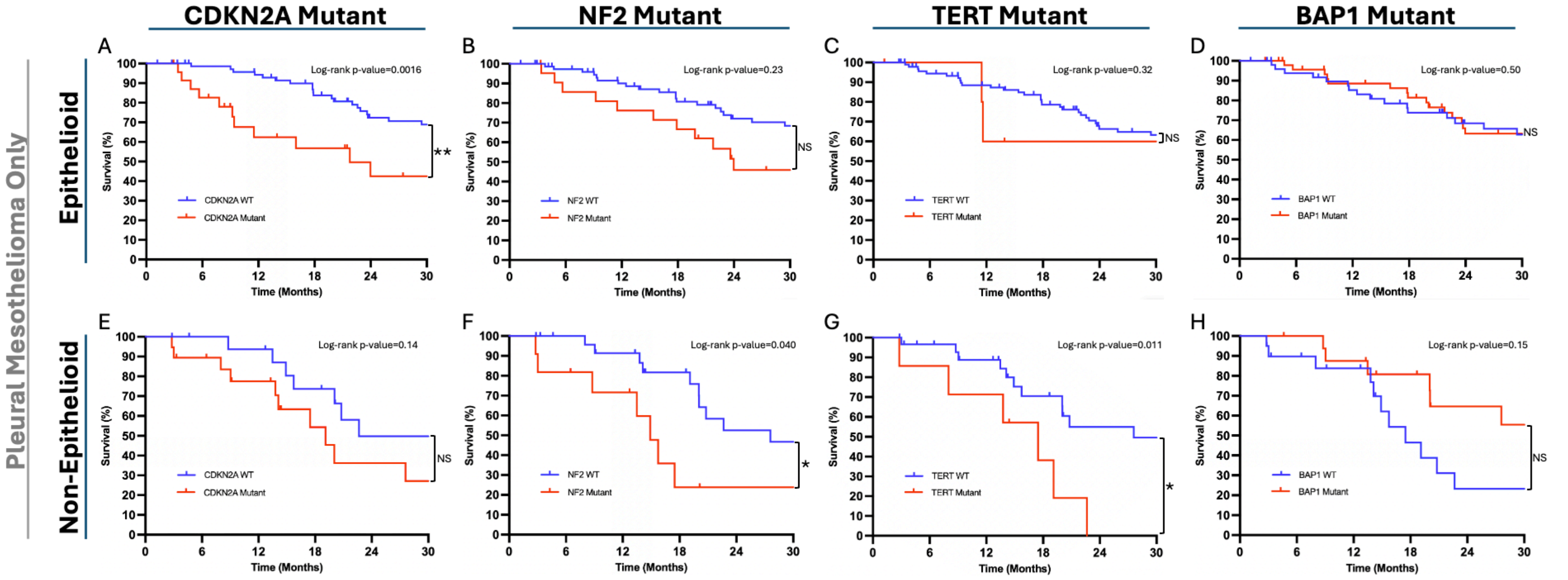

<u>Supplemental Figure 3:</u> <u>Overall Survival for Pleural Mesothelioma Patients by Histologic Subtype and Select Somatic Mutations.</u> Overall survival (OS) was compared among pleural mesothelioma patients by histologic subtypes including epithelioid and non-epithelioid (biphasic or sarcomatoid). Among pleural mesothelioma patients with epithelioid subtype, OS was compared for the presence versus absence of key somatic mutations including **(a)** *CDKN2A*, **(b)** *NF2*, **(c)** *TERT*, and **(d)** *BAP1*. OS analysis was also conducted for pleural mesothelioma patients with non-epithelioid subtypes for these same mutations including **(e)** *CDKN2A*, **(f)** *NF2*, **(g)** *TERT*, and **(h)** *BAP1*.

Group	Subgroup	Patient	Full Variant Description	Variant Type	Variant Effect	Truncating Mutation	Mutant Amino Acid Position	Early Stop Codon Position	Percent of Protein Truncated (lost)	Site of Protein Domain Truncation
Pathogenic Mutations	NF2 Truncation Variants	Patient A:	NF2 c.1396C>T, p.R466*	C>T	Non-sense	Yes (non-sense)	466	466	22%	Central α-Helical Domain
		Patient B:	NF2 c.241del, p.V81Yfs*42	Small Deletion (INDEL)	Frameshift and Early Stop	Yes (frameshift+early stop codon)	241	283	52%	FERM F3 Domain
		Patient C:	NF2 c.1021C>T, p.R341*	C>T	Non-sense	Yes (non-sense)	341	341	43%	Early α-Helical Domain
		Patient D:	NF2 c.784C>T, p.R262*	C>T	Non-sense	Yes (non-sense)	262	262	56%	FERM F3 Domain
		Patient E:	NF2 c.61_67del, p.T21Pfs*2	Small Deletion (INDEL)	Frameshift and Early Stop	Yes (frameshift+early stop codon)	21	23	96%	FERM F1 Domain
		Patient F:	NF2 c.834_867del, p.K278Nfs*7	Small Deletion (INDEL)	Frameshift and Early Stop	Yes (frameshift+early stop codon)	278	285	52%	FERM F3 Domain
		Patient G:	NF2 c.970C>T, p.Q324*	C>T	Non-sense	Yes (non-sense)	324	324	46%	Early α-Helical Domain
		Patient H:	NF2 c.949G>T, p.E317*	G>T	Non-sense	Yes (non-sense)	317	317	47%	Early α-Helical Domain
		Patient I:	NF2 c.1009C>T, p.Q337*	C>T	Non-sense	Yes (non-sense)	337	337	43%	Early α-Helical Domain
		Patient J:	NF2 c.193C>T, p.Q65*	C>T	Non-sense	Yes (non-sense)	65	65	89%	FERM F1 Domain
		Patient K:	NF2 c.970del, p.Q324Rfs*22	Small Deletion (INDEL)	Frameshift and Early Stop	Yes (frameshift+early stop codon)	324	346	42%	Early α-Helical Domain
		Patient L:	NF2 c.702_732del, p.G235Tfs*6	Small Deletion (INDEL)	Frameshift and Early Stop	Yes (frameshift+early stop codon)	235	241	59%	FERM F3 Domain
		Patient M:	NF2 c.1188del, p.K396Nfs*30	Small Deletion (INDEL)	Frameshift and Early Stop	Yes (frameshift+early stop codon)	396	426	28%	Central α-Helical Domain
		Patient N:	NF2 c.449_468del, p.T150Cfs*46	Small Deletion (INDEL)	Frameshift and Early Stop	Yes (frameshift+early stop codon)	150	196	67%	FERM F2 Domain
		Patient O:	NF2 c.737del, p.P246Lfs*5	Small Deletion (INDEL)	Frameshift and Early Stop	Yes (frameshift+early stop codon)	246	251	58%	FERM F3 Domain
		Patient P:	NF2 c.616G>T, p.E206*	G>T	Non-sense	Yes (non-sense)	206	206	65%	FERM F3 Domain
	NF2 Other Variants	Patient Q:	NF2 Loss	Large Deletion	Gene Loss	Not necessarily (gene loss)	–	–	–	–
		Patient R:	NF2 Rearrangement	Rearrangement	Structural Breakdown/Gene Loss	Not necessarily (gene loss)	–	–	–	–
		Patient S:	NF2 c.246_272del, p.D83_P91del	Small Deletion (INDEL)	In-frame Deletion in critical FERM Domain	No (in-frame deletion)	–	–	–	–
		Patient T:	NF2 c.448-2A>C, p.?	A>C	Canonical Splice-site Disruption	Not necessarily (splicing variant)	–	–	–	–
		Patient U:	NF2 c.771_810+5del, p.?	Small Deletion (INDEL)	Canonical Splice-site Disruption	Not necessarily (splicing variant)	–	–	–	–
		Patient V:	NF2 Loss	Large Deletion	Gene Loss	Not necessarily (gene loss)	–	–	–	–
		Patient W:	NF2 c.276_299del, p.T93_F100del	Small Deletion (INDEL)	In-frame Deletion in critical FERM Domain	No (in-frame deletion)	–	–	–	–
		Patient X:	NF2 Loss	Large Deletion	Gene Loss	Not necessarily (gene loss)	–	–	–	–
		Patient Y:	NF2 Loss	Large Deletion	Gene Loss	Not necessarily (gene loss)	–	–	–	–
		Patient Z:	NF2 Loss	Large Deletion	Gene Loss	Not necessarily (gene loss)	–	–	–	–
		Patient A2	NF2 c.1340+1G>T, p.?	G>T	Canonical Splice-site Disruption	Not necessarily (splicing variant)	–	–	–	–
		Patient B2	NF2 Loss	Large Deletion	Gene Loss	Not necessarily (gene loss)	–	–	–	–
		Patient C2	NF2 c.138_161del, p.F47_L54del	Small Deletion (INDEL)	In-frame Deletion in critical FERM Domain	No (in-frame deletion)	–	–	–	–
	NF2 VUS	Patient D2	NF2 c.655G>A, p.V219M	G>A	Missense not in FERM Domain	No (missense)	–	–	–	–
		Patient E2	NF2 c.114G>A, p.E38E	G>A	Silent Mutation	No (silent mutation)	–	–	–	–

Supplemental Figure 4: Hybrid Oncoplot with Full Mutational Reports of Patients with *NF2*-Mutant Pleural Mesothelioma. This figure is a continuation of **Figure 6** and shows the complete mutational report information for all patients with pathogenic *NF2* mutant pleural mesothelioma. It follows the same patient groups as shown in **Figure 6** but also includes significantly more detail on the specific pathogenic variants and classifications following the literature guidelines.[48,49]

Supplemental Tables:

Supplemental Table 1: Tumor Mutational Profiles for Less Common Mutations			
		Number of Patients (% of all patients)	
	**All Patients**	**Pleural Mesothelioma**	**Peritoneal Mesothelioma**
**Tumor Somatic Mutational Profiling**	N = 199*	N = 137	N = 58
Tumor Mutations:			
ASXL1	1 (0.5%)	1 (0.7%)	0 (0%)
ATR	1 (0.5%)	1 (0.7%)	0 (0%)
ATRX	1 (0.5%)	1 (0.7%)	0 (0%)
B2M	1 (0.5%)	1 (0.7%)	0 (0%)
BRCA1	1 (0.5%)	1 (0.7%)	0 (0%)
CSF1R	1 (1.6%)	0 (0%)	1 (1.7%)
CSF3R	1 (0.5%)	1 (0.7%)	0 (0%)
CUX1	1 (0.5%)	1 (0.7%)	0 (0%)
DDX41	1 (0.5%)	1 (0.7%)	0 (0%)
DNMT3A	1 (0.5%)	1 (0.7%)	0 (0%)
EP300	1 (0.5%)	1 (0.7%)	0 (0%)
EPHA5	1 (1.6%)	0 (0%)	1 (1.7%)
GNAQ	1 (0.5%)	1 (0.7%)	0 (0%)
H3F3A	1 (0.5%)	1 (0.7%)	0 (0%)
HNF1A	1 (0.5%)	1 (0.7%)	0 (0%)
KDM6A	1 (0.5%)	1 (0.7%)	0 (0%)
KDR	1 (1.7%)	0 (0%)	1 (1.7%)
KRAS	1 (0.5%)	1 (0.7%)	0 (0%)
MET	1 (0.5%)	1 (0.7%)	0 (0%)
MLH1	1 (0.5%)	1 (0.7%)	0 (0%)
MLH3	1 (0.5%)	1 (0.7%)	0 (0%)
MRE11A	1 (0.5%)	1 (0.7%)	0 (0%)
PALB2	1 (0.5%)	1 (0.7%)	0 (0%)
PIK3CA	1 (0.5%)	1 (0.7%)	0 (0%)
RAD51	1 (0.5%)	1 (0.7%)	0 (0%)
RB1	1 (0.5%)	1 (0.7%)	0 (0%)
STAG2	1 (1.6%)	0 (0%)	1 (1.7%)
TSC	1 (0.5%)	1 (0.7%)	0 (0%)
CTNNA1†	1 (25%)	0 (0%)	0 (0%)
STK11†	1 (25%)	0 (0%)	0 (0%)

*Includes four patients with bicavitary (both pleural and peritoneal) mesothelioma involvement.

Boldface entries indicate statistical significance (p-value < 0.05). χ^2 test or Fisher's exact test for categorical variables (number (%)).

†Mutations only tested in bicavitary mesothelioma patients.

Supplemental Table 1: Tumor Mutational Profiles for Less Common Gene Mutations for Pleural versus Peritoneal Mesothelioma. **Supplemental Table 1** is an extension of the tumor mutations section in **Table 1** which together show the complete list of tested mutations.

Supplemental Table 2: Tumor Mutational Profiles for Epithelioid Histologic Subtype Only			
	Epithelioid Histologic Subtype		
Tumor Somatic Mutational Profiling	Pleural Mesothelioma N = 99	Peritoneal Mesothelioma N = 54	p-value
Tumor Mutations:§			
BAP1	48 (48%)	28 (52%)	0.7
CDKN2A	24 (24%)	7 (13%)	0.1
NF2	22 (22%)	10 (19%)	0.6
TP53	29 (29%)	5 (9.3%)	**0.004**
TERT	6 (6.1%)	4 (7.4%)	0.7
DDX3X	5 (5.1%)	5 (9.3%)	0.3
PBRM1	3 (3.0%)	7 (13%)	**0.034**
FBXW7	3 (3.0%)	0 (0%)	0.6
CHEK2	2 (2.0%)	1 (1.9%)	>0.9
PTEN	3 (3.0%)	1 (1.9%)	>0.9
SETD2	4 (4.0%)	0 (0%)	0.3
ATM	1 (1.0%)	2 (3.7%)	0.3
NF1	1 (1.0%)	1 (1.9%)	>0.9
ARID2	2 (2.0%)	0 (0%)	0.5
MSH6	1 (1.0%)	1 (1.9%)	>0.9
BRCA2	0 (0%)	0 (0%)	-
FAT3	1 (1.0%)	1 (1.9%)	>0.9
MDM2	0 (0%)	0 (0%)	-
NRAS	1 (1.0%)	0 (0%)	>0.9
WT1	1 (1.0%)	1 (1.9%)	>0.9

Tumor Mutations§ shown for all mutations with ≥2 occurrences among the entire patient cohort regardless of histologic subtype.
Boldface entries indicate statistical significance (p-value < 0.05). χ^2 test or Fisher's exact test for categorical variables (number (%)).

Supplemental Table 2: Tumor Mutational Profiles of Epithelioid Histologic Subtype for Pleural vs. Peritoneal Mesothelioma.

Supplemental Table 3: Tumor Mutational Profiles for Pleural Mesothelioma Only by Histologic Subtype

Tumor Somatic Mutational Profiling	Pleural Mesothelioma			p-value‡
	**Biphasic** N = 30	**Sarcomatoid** N = 8	**Epithelioid** N = 99	
Tumor Mutations:§				
BAP1	15 (50%)	2 (25%)	48 (48%)	0.7
CDKN2A	12 (40%)	7 (88%)	24 (24%)	**0.004**
NF2	9 (30%)	3 (38%)	22 (22%)	0.3
TP53	7 (23%)	1 (13%)	29 (29%)	0.3
TERT	2 (6.7%)	5 (63%)	6 (6.1%)	**0.046**
DDX3X	1 (3.3%)	1 (13%)	5 (5.1%)	>0.9
PBRM1	0 (0%)	0 (0%)	3 (3.0%)	0.6
FBXW7	1 (3.3%)	1 (13%)	3 (3.0%)	0.6
CHEK2	2 (6.7%)	0 (0%)	2 (2.0%)	0.3
PTEN	1 (3.3%)	0 (0%)	3 (3.0%)	>0.9
SETD2	1 (3.3%)	0 (0%)	4 (4.0%)	>0.9
ATM	0 (0%)	0 (0%)	1 (1.0%)	>0.9
NF1	1 (3.3%)	0 (0%)	1 (1.0%)	0.5
ARID2	1 (3.3%)	0 (0%)	2 (2.0%)	>0.9
MSH6	1 (3.3%)	0 (0%)	1 (1.0%)	0.5
BRCA2	2 (6.7%)	0 (0%)	0 (0%)	0.075
FAT3	0 (0%)	0 (0%)	1 (1.0%)	>0.9
MDM2	1 (3.3%)	1 (13%)	0 (0%)	0.075
NRAS	0 (0%)	1 (13%)	1 (1.0%)	0.5
WT1	0 (0%)	0 (0%)	1 (1.0%)	>0.9

Tumor Mutations§ shown for all mutations with ≥2 occurrences among the entire patient cohort regardless of histologic subtype.

p-value‡ comparing characteristics of pleural mesothelioma patients with epithelioid versus non-epithelioid histologic subtype. Non-epithelioid is defined as either biphasic or sarcomatoid histologic subtype.

Boldface entries indicate statistical significance (p-value < 0.05). χ^2 test or Fisher's exact test for categorical variables (number (%)).

Supplemental Table 3: Tumor Mutational Profiles for Pleural Mesothelioma Histologic Subtypes.

Supplemental Table 4: Comparison of Patients with Shortened versus Extended Survival

Patient Characteristics	Number of Patients (% of all patients) >5 year OS N = 20	<1 year OS N = 21	p-value‡
**Demographics and Medical History**			
Age at Diagnosis, years (SD)	60 (14)	70 (8)	**0.01**
Sex:			0.7
Female	13 (65%)	15 (71%)	
Male	7 (35%)	6 (29%)	
White	19 (95%)	20 (95%)	>0.9
Asbestos Exposure	10 (50%)	12 (57%)	0.6
History of Other Cancer	2 (10%)	4 (19%)	0.7
**Treatment Received**			
Treatment Type:			**<0.001**
Palliative Treatment Only	18 (90%)	2 (11%)	
Non-Palliative Treatment	2 (10%)	17 (89%)	
Surgery	18 (90%)	2 (9.5%)	**<0.001**
Immunotherapy	11 (55%)	7 (33%)	0.2
Neoadjuvant Chemotherapy	2 (10%)	1 (4.8%)	0.6
Adjuvant Chemotherapy	12 (60%)	1 (4.8%)	**<0.001**
Palliative Chemotherapy	14 (70%)	16 (76%)	0.7
**Pathologic Findings**			
Mesothelioma Anatomic Site:			0.14
Pleural	12 (60%)	17 (81%)	
Peritoneal	8 (40%)	4 (19%)	
**Tumor Somatic Mutational Profiling**			
Tumor Mutational Burden, mean (SD)	1.50 (0.74)	2.14 (0.99)	**0.019**
Presence of Any Tumor Mutation	18 (90%)	20 (95%)	0.6
Tumor Mutations:§			
BAP1	13 (65%)	8 (38%)	0.085
CDKN2A	1 (5.0%)	14 (67%)	**<0.001**
NF2	2 (10%)	9 (43%)	**0.018**
TP53	2 (10%)	4 (19%)	0.7
TERT	2 (10%)	5 (24%)	0.4
DDX3X	3 (15%)	0 (0%)	0.11
PBRM1	1 (5.0%)	0 (0%)	0.5
FBXW7	0 (0%)	0 (0%)	-
CHEK2	0 (0%)	0 (0%)	-
PTEN	2 (10%)	1 (4.8%)	0.6
SETD2	1 (5.0%)	0 (0%)	0.5
ATM	1 (5.0%)	0 (0%)	0.5
NF1	0 (0%)	1 (4.8%)	>0.9
ARID2	0 (0%)	0 (0%)	-
MSH6	1 (5.0%)	0 (0%)	0.5

BRCA2	1 (5.0%)	0 (0%)	0.5
FAT3	0 (0%)	0 (0%)	-
MDM2	0 (0%)	1 (4.8%)	>0.9
NRAS	0 (0%)	0 (0%)	-
WT1	0 (0%)	0 (0%)	-

p-value‡ comparing characteristics of patients with <1 year OS versus patients with >5 year OS from date of mesothelioma diagnosis.
Boldface entries indicate statistical significance (p-value < 0.05).
†Kadota System for scoring of variables.
Tumor Mutations§ shown for all mutations with ≥2 occurrences among the entire patient cohort.
χ2 test or Fisher's exact test for categorical variables (number (%)) and t-test for continous variables (mean(standard deviation)).

Supplemental Table 4: Comparison of Patients with Shortened versus Extended Survival. Overview of patient characteristics including patient demographics, treatments, pathology, outcomes, and somatic mutation profiles with comparisons of characteristics for patients with >5 year survival versus <1 year survival from date of mesothelioma diagnosis.
